# Effects of salinomycin and niclosamide on small cell lung cancer and small cell lung cancer circulating tumor cell lines

**DOI:** 10.1007/s10637-019-00847-8

**Published:** 2019-08-24

**Authors:** Maximilian Hochmair, Barbara Rath, Lukas Klameth, Ernst Ulsperger, Christoph Weinlinger, Andreas Fazekas, Adelina Plangger, Robert Zeillinger, Gerhard Hamilton

**Affiliations:** 1 Respiratory Oncology Unit, Otto Wagner Hospital, Baumgartner Höhe, Vienna, Austria; 2 Department of Surgery, Medical University of Vienna, Spitalgasse, Vienna, Austria; 3 Department of Pathophysiology and Allergy Research, Medical University of Vienna, Vienna, Austria; 4 Hospital Horn, Spitalgasse, Horn, Austria; 5 Department of Gynecology and Obstetrics, Molecular Oncology Group, Medical University of Vienna, Vienna, Austria

**Keywords:** Small cell lung cancer, Circulating tumor cells, Cancer stem cells, Salinomycin, Niclosamide, CD133, Cytotoxicity

## Abstract

Tumor dissemination and recurrence is attributed to highly resistant cancer stem cells (CSCs) which may constitute a fraction of circulating tumor cells (CTCs). Small cell lung cancer (SCLC) constitutes a suitable model to investigate the relation of CTCs and CSCs due to rapid tumor spread and a high number of CTCs. Expansion of five SCLC CTC lines (BHGc7, 10, 16, 26 and UHGc5) in vitro at our institution allowed for the analysis of CSC markers and cytotoxicity of the CSC-selective drugs salinomycin and niclosamide against CTC single cell suspensions or CTC spheroids/ tumorospheres (TOS). Salinomycin exerted dose-dependent cytotoxicity against the SCLC lines but, with exception of BHGc7 TOS, there was no markedly enhanced activity against TOS. Similarly, niclosamide exhibits high activity against BHGc7 TOS and UHGc5 TOS but not against the other CTC spheroids. High expression of the CSC marker CD133 was restricted to three SCLC tumor lines and the BHGc10 CTC line. All SCLC CTCs are CD24-positive but lack expression of CD44 and ABCG2 in contrast to the SCLC tumor lines which show a phenotype more similar to that of CSCs. The stem cell marker SOX2 was found in all CTC lines and SCLC GLC14/16, whereas elevated expression of Oct-3/4 and Nanog was restricted to BHGc26 and UHGc5. In conclusion, the SCLC CTCs established from patients with relapsed disease lack a typical CSC phenotype in respect to chemosensitivity to CSC-selective drugs, surface markers, expression of pluripotent stem cell and transcription factors.

## Introduction

The high mortality rate of cancer patients is due to the refractoriness of metastatic cells to current treatments. Tumors seem to contain a small subpopulation of cancer stem cells (CSCs) which sustain primary tumors and metastases [1–4]. CSCs are chemoresistant due to efficient DNA damage repair, the activation of survival pathways, decreased apoptosis, immune evasion and the adaptation to a hostile microenvironment [5]. Therefore, conventional chemotherapy eliminates the bulk of the tumor cells but CSCs survive and reconstitute the tumor as well as metastases [6]. Tumor dissemination is accomplished by circulating tumor cells (CTCs) which may show CSCs characteristics provided that these cells play a decisive role in cancer metastasis [7]. Thus, targeting CSCs in combination with standard chemotherapy may improve outcomes of cancer patients and procure long-lasting responses. However, attempts to target and eliminate CSC by suitable drugs were clinically not successful so far [8]. CSCs have been identified in most solid tumors and this subpopulation has been found to be enriched after chemotherapy [4, 9–12]. For most solid tumors, expression of CD133, CD44, CD24, CD166, epithelial cell adhesion molecule (EpCAM), aldehyde dehydrogenases (ALDH) and ATP-binding cassette subfamily G member 2 (ABCG2) have been proposed as selective markers [9, 10, 13–15]. Furthermore, altered developmental pathways such as Notch, Wnt and Hedgehog drive CSC growth, progression and drug resistance [16, 17]. Additionally, CSCs express pluripotency transcription factors OCT4, Nanog and Sox2 and show enhanced tumor-sphere formation [18–20]. Increased oxidative phosphorylation OXPHOS is a hallmark of resistance to chemotherapy and, therefore, the OXPHOS inhibitor salinomycin was shown to selectively kill CSCs [21–24]. CD133^+^ colorectal CSC-like cells were sensitive to salinomycin treatment, but not to the conventional anticancer drug oxaliplatin [25]. A few clinical case reports have documented effectiveness of salinomycin in therapy-resistant cancer patients, e.g., a patient with metastatic invasive ductal breast cancer treated with salinomycin showed induction of clinical tumor regression [26, 27]. In particular, salinomycin-induced ER Ca^2+^ depletion inhibits Wnt signaling by down-regulating β-catenin [28, 29]. Additionally, other drugs such as niclosamide, effectively inhibit activation of the Wnt/β-catenin signaling and reduce the formation of primary and secondary tumorspheres [30]. The anthelminthic niclosamide showed similar effects as salinomycin on cancer stemness as well as uncoupling of mitochondrial phosphorylation [31].

Metastasis-initiating capabilities of CTCs based on stemness properties are difficult to detect since CTCs show extensive heterogeneity and only an extremely small fraction of these cells is able to establish secondary lesions [32, 33]. In vitro expansion of relevant CTCs has been reported for only a limited number of tumors and cell lines so far [34]. We have obtained five permanent CTC cell lines from blood samples of patients bearing extended disease small cell lung cancer (ED-SCLC) [35]. Small cell lung cancer (SCLC) comprises approximately 15% of all lung cancers and is found disseminated in the great majority of patients at first presentation [36]. Patients respond well to first-line platinum-based combination therapy with response rates on the order of 70%–90% in limited disease and 50%– 60% in extended disease [36, 37]. However, nearly all patients with SCLC eventually relapse with chemo- and radioresistant tumors which are difficult to treat and have a dismal prognosis [38]. These characteristics of SCLC suggest that it may be enriched in CSCs and the general resistance is effected by a CSC subpopulation [39, 40]. In the present study, the SCLC CTC and tumor cell lines were used to study chemosensitivity to the CSC-targeting drugs salinomycin and niclosamide and to characterize their expression of selected CSC markers and pluripotent transcription factors.

## Methods

### Cell lines and reagents

SCLC26A was established in our lab from a pleural effusion of a SCLC patient before treatment. GLC14 and GLC16 have been derived from the same patient before and after first cycles of chemotherapy [41, 42], DMS153 and DMS273 were cultured from liver metastases of pretreated patients and NCI-H526 from a bone metastasis, respectively, whereas NCI-H417, DMS53, DMS114 and NCI-H69 were established from primary SCLCs before treatment. All cell lines, with exception of SCLC26A and HEK293 (ATCC, Manassas, VA, USA), were obtained from the Finsen Center, Copenhagen, Denmark. The SCLC CTC cell lines BHGc7, 10, 16, 26 and UHGc5 were established from blood samples of ED-SCLC patients at our institution [35, 43, 44 and unpublished]. Single cell suspension of the CTC lines form tumorospheres in regular tissue culture spontaneously. Blood collection and generation of cell lines was done according to the Ethics Approval 366/2003 by the Ethics Committee of the Medical University of Vienna, Vienna, Austria. Cells were cultured in RPMI-1640 medium (Seromed, Berlin, Germany) supplemented with 10% fetal bovine serum (Seromed) and antibiotics (Sigma-Aldrich, St. Louis, MO, USA). Salinomycin and niclosamide were obtained from Sigma-Aldrich.

### Light microscopy

Pictures of clusters of SCLC CTCs were acquired from tissue cultures and processed using Photoshop (Adobe, San Jose, CA, USA). Preparation of a TOS histological section was done as described previously [35].

### Cytotoxicity assays

1 × 10^4^ cells in form of single cells or tumorospheres (TOS) in 100 μl medium were distributed to wells of 96-well microtiter plates (TPP, Trasadingen Switzerland) and ten 2-fold dilutions of the test compounds were added from stock dilutions as described [35]. Assays were at least performed in triplicate. The plates were incubated for four days under tissue culture conditions and viable cells detected using a modified MTT assay (EZ4U, Biomedica, Vienna, Austria). The respective dilutions of the compounds tested are present for the whole incubation period. IC_50_ values were determined from dose-response curves using Origin 9.1 software (OriginLab, Northampton, MA, USA).

### Flow cytometry analysis

Antibodies were obtained from Biolegend (San Diego, CA, USA). Expression of CD133 (Clone 315-2C11), CD24 (ML5), CD44 (IM7) and ABCG2 (5D3) was determined in flow cytometry using corresponding secondary reagents (Sigma-Aldrich) and a Cytomics FC500 flow cytometer (Beckman-Coulter, Brea, CA, USA) in indirect immunofluorescence. Data analysis and histogram overlays were done employing the Kaluza flow analysis software (Beckman Coulter).

### Western blot Array

Pluripotent stem cell markers were analyzed using the ARY010 Proteome Profiler Array (R&D Systems, Minneapolis, MN, USA) according to manufacturer’s instructions. Experiments were done in duplicate and the different tests were calibrated using the six reference spots included for each individual membrane. Arrays were evaluated using ImageJ and Origin 9.1 software (OriginLab, Northampton, MA, USA).

### Statistical analysis

Statistical significance was tested by t-tests and *p* < 0.05 regarded as significant difference using Origin software (Originlab, Northampton, MA, USA).

## Results

### Chemosensitivity of the SCLC cell lines to salinomycin

**Fig. 1:**
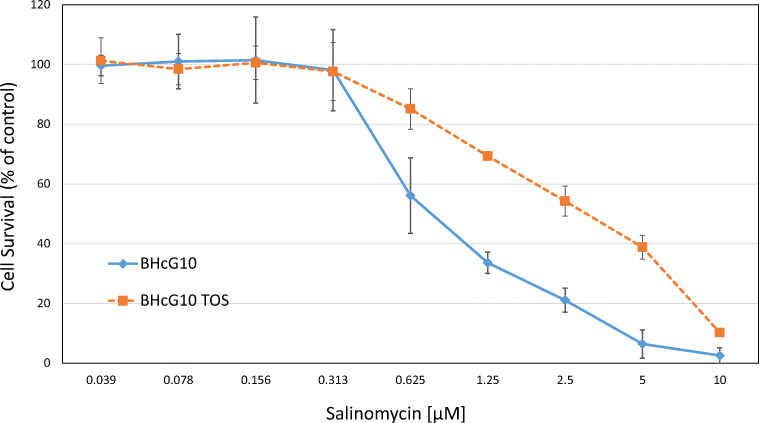
Salinomycin dose-response curves for BHGc10 single cells and BHGc10 TOS. Salinomycin was applied in 9 2-fold dilution steps and cell survival determined in a MTT assay. Values are presented as mean ± SD

The cytotoxic activity of salinomycin against SCLC CTC and tumor cell lines was tested in MTT proliferation assays. An example of a dose-response curve for BHGc10 single cells and TOS is shown in Fig. 1. BHGc10 cells in the multicellular TOS are approximately 2.5 times more resistant to salinomycin compared to the corresponding single cell suspension tested.

**Fig. 2:**
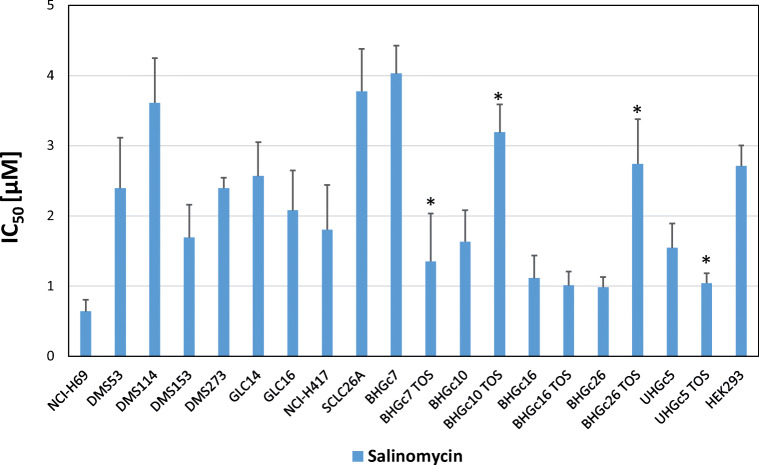
IC_50_ values for salinomycin cytotoxicity against SCLC cell lines**.** Dose-response curves were obtained as shown in Fig. 1. and IC_50_ values calculated by curve fitting (mean ± SD) of at least three independent MTT assays. Significant differences in IC_50_ values between SCLC CTC lines as single cells and TOS are indicated by an asterisk

The IC_50_ values for all SCLC cell lines are shown in Fig. 2, in case of the CTC lines for single cell suspensions and TOS, respectively. NCI-H69, BHGc16/TOS, BHGc26 and UHGc5 exhibited the lowest IC_50_ values in the 1 μM range. All other SCLC lines yield IC_50_ values for salinomycin in the 2–4 μM range, including BHGc7 single cells, BHGc10 and BHGc26TOS.

### Chemosensitivity of the SCLC cell lines to niclosamide

**Fig. 3:**
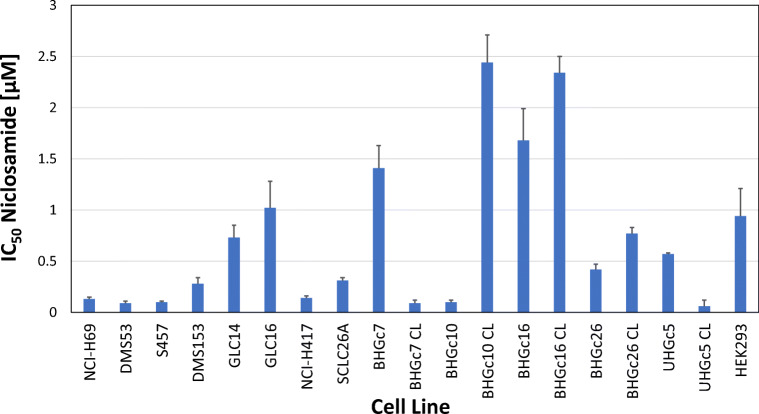
IC_50_ values for niclosamide cytotoxicity assays of SCLC cell lines. Dose-response curves were obtained as shown in Fig. 1. and IC_50_ values obtained by curve fitting (mean ± SD) of at least three independent MTT assays. Significant differences in IC_50_ values between SCLC CTC lines as single cells and TOS are indicated by an asterisk

The cytotoxic activity of niclosamide against CTC and tumor SCLC cell lines was tested in MTT proliferation assays (Fig. 3). The cell lines exhibited variable chemosensitivity to niclosamide and, in particular, CTC tumorospheres were either more sensitive to the drug as in case of BHGc7 and UHGc5, or more resistant, as for BHGc10, BHGc16 and BHGc26, compared to the same cell lines as single cell suspensions.

### Expression of CSC markers by SCLC-derived CTCs and tumor cell lines

**Fig. 4:**
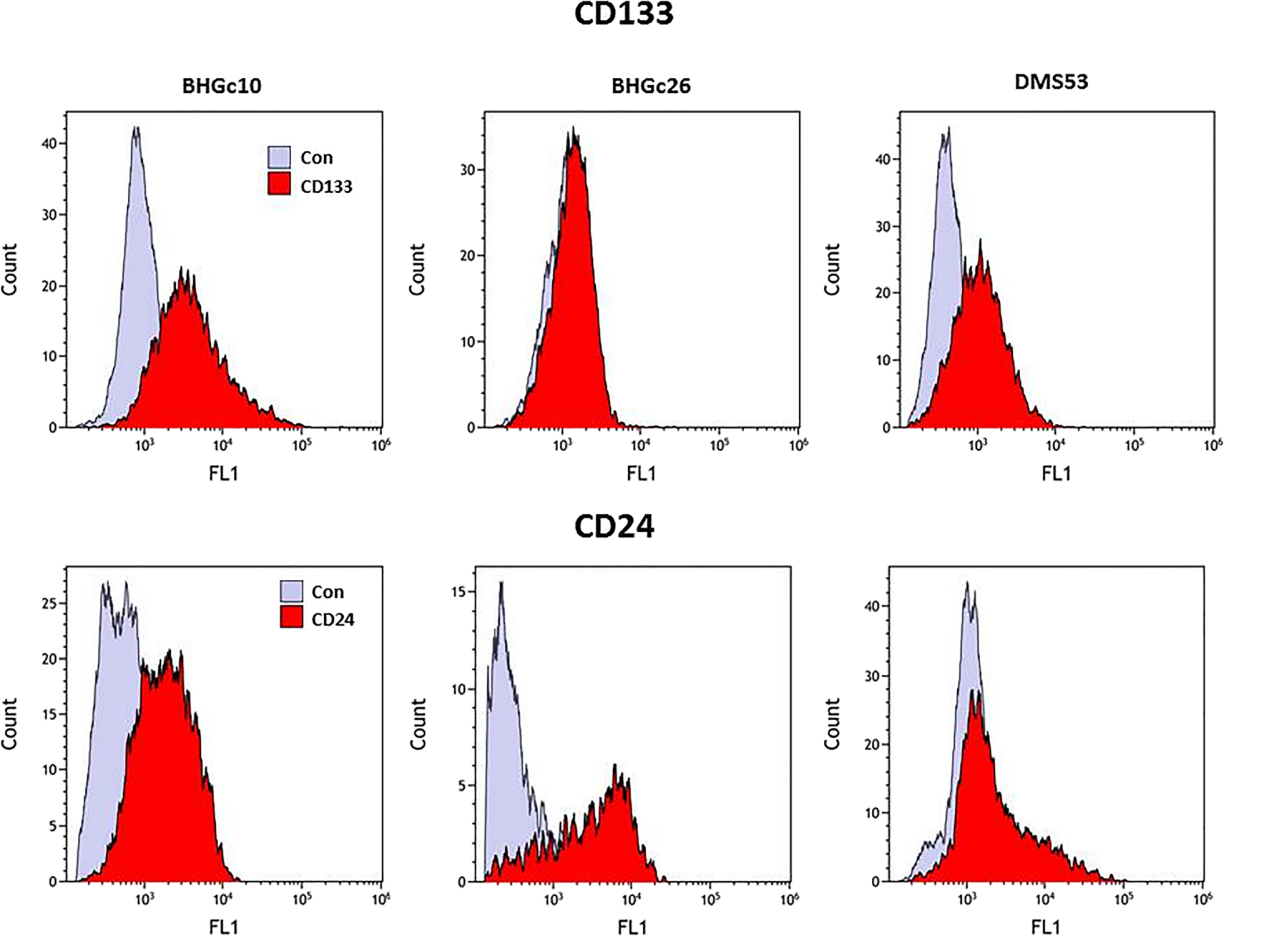
Flow cytometric histograms of CD133 and CD24 expression of SCLC cells Examples of the histogram overlays show fluorescence labeling of BHGc10, BHGc26 and DMS53 SCLC cell lines with antibodies to CD133, CD24, and controls respectively

Analysis of CSC marker expression was done using indirect immunofluorescence and flow cytometry. High expression of the CSC marker CD133 was detected in the BHGc10 CTC and DMS53 tumor cell lines, whereas expression was absent in the BHGc16 CTC cells (Fig. 4).

**Fig. 5:**
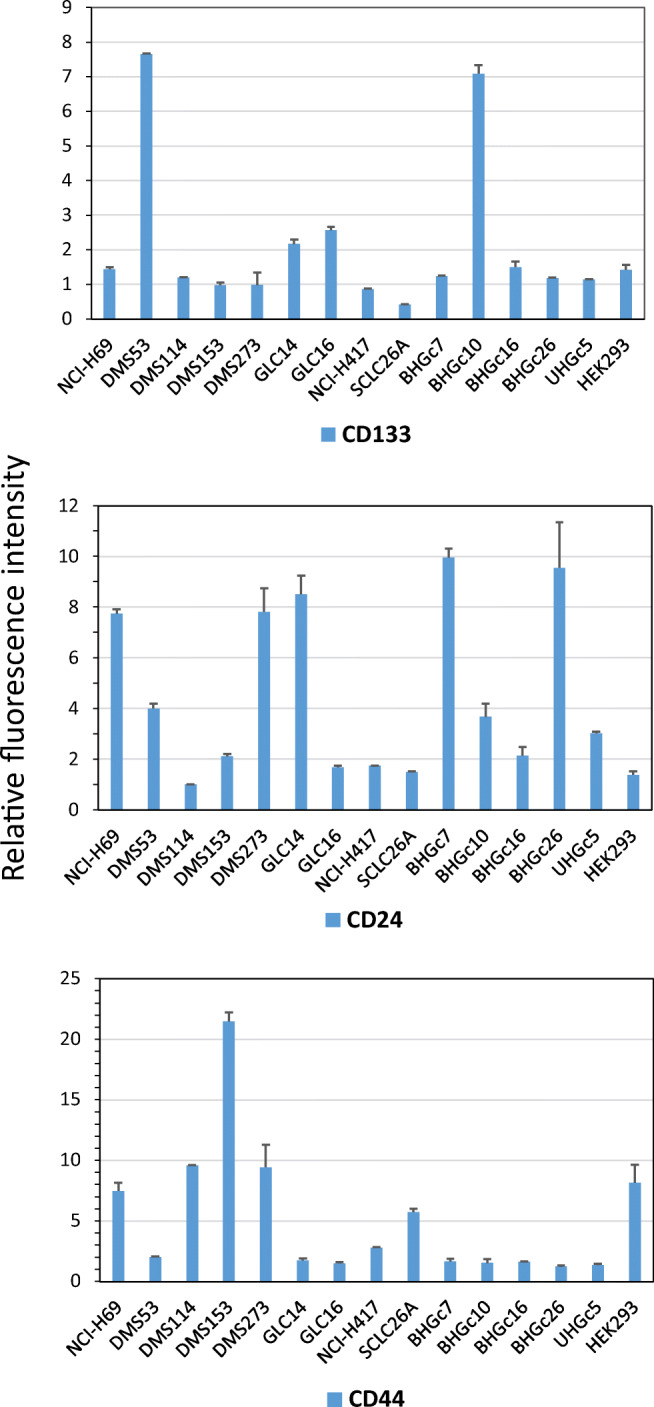
Expression profiles of CD133, CD24 and CD44 of the SCLC cell lines. Results are shown as relative fluorescence intensities (mean peak intensity specific fluorescence/mean peak intensity of isotype controls; mean values ± SD) obtained in indirect immunofluorescence labeling of the cell lines with antibodies directed to CD133, CD24 and CD44, respectively

These three SCLC cell lines showed expression of CD24 to a variable degree In addition to CD133-positive DMS53 and BHGc10, significant expression of this antigen in GLC14 and GLC16 SCLC lines and peak labeling of BHGc16 CTC and NCI-H69 cells was found (Fig. 5).

CD24 was significantly expressed in the CTC SCLC cell lines, as well in NCI-H69, DMS53, DMS273, GLC14 tumor cells with exception of DMS114 (Fig. 5). In contrast, CD44 was expressed in NCI-H69, DMS114, DMS153 and DMS273 cells with weak labeling of SCLC26A and HEK293 cell lines, whereas for all CTC SCLC lines this antigen was found at low levels. None of the CTC lines stained positively for ABCG2 (data not shown).

### Expression of the pluripotent stem cell markers in SCLC cell lines

**Fig. 6:**
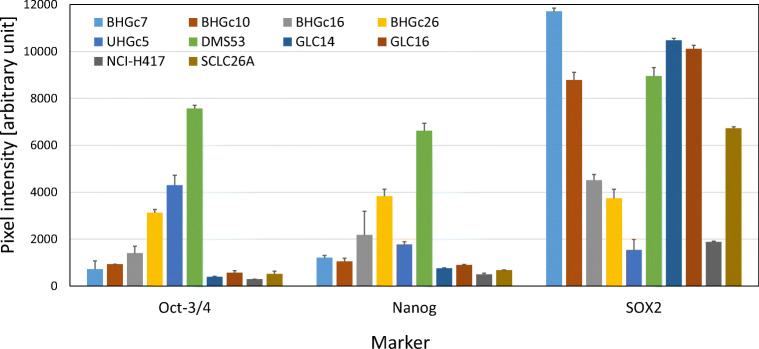
Expression of stem cell markers Oct3/4, Nanog and SOX2 in selected SCLC cell lines. Values represent arbitrary pixel intensities (mean ± SD) obtained from analysis of Western blot arrays. Individual membranes are calibrated using the internal controls

Expression of selected pluripotent stem cell markers, Oct-3/4, Nanog and SOX2 were determined using human Profiler Western blot arrays (Fig. 6). Among the SCLC CTC cell lines, Oct-3/4 showed higher expression in BHGc26 and UHGc5 cells and Nanog in BHGc26 cells, whereas BHGc7, 10 and 16 showed low antigen levels. Both antigens are highly expressed in DMS53 but not in other SCLC lines. As expected, SOX2 is highly expressed in all SCLC/CTC lines, with exception of UHGc5 and NCI-H417 which showed lower expression.

### Light microscopy of BHGc26 TOS and tissue section

**Fig. 7:**
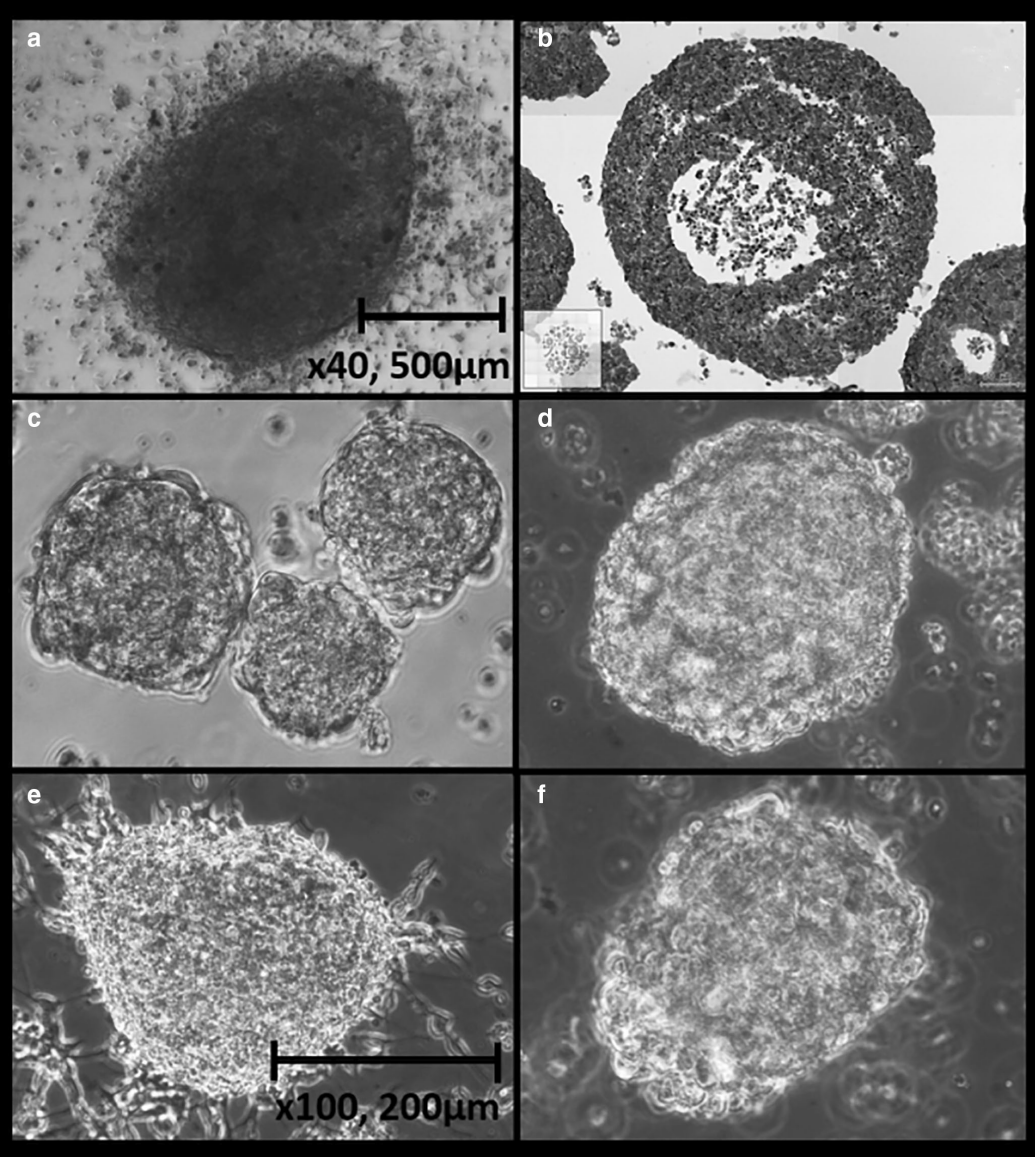
Light microscopy of SCLC CTC TOS and a BHGc26 TOS histological section. BHGc7 grows as large cluster on a layer of adherent cells (7A). A histological section of the BHGc26 TOS shows the typical outer rim of intact and proliferating cells and the necrotic core (Fig. 7b). The histological picture indicates the insufficient oxygen and nutrients supply for the inner layers of such large spheroidal structures which generates a hypoxic niche for the CTCs. The typical large tumorospheres of BHGc10, 16, 26 and UHGc5 are shown in Fig. 7c-f, respectively

Light microscopic pictures of the SCLC CTC TOS are shown in Fig. 7. BHGc7 TOS develop as large spheroids on a layer of adherent cells whereas the other SCLC CTC TOS exist as large spheroidal structures floating in tissue culture medium. In contrast to other spheroid cultures published, the CTC TOS form spontaneously without the need of stirring or low-attachment culture conditions. A histological section of the BHGc26 TOS shows the typical outer rim of intact and proliferating cells and the necrotic core (Fig. 7b). The histological picture indicates the insufficient oxygen and nutrients supply for the inner layers of such large spheroidal structures which generates a hypoxic niche for a fraction of the CTCs.

## Discussion

In the present study, the cytotoxic activities of the CSC-selective drugs salinomycin and niclosamide were tested against SCLC and SCLC CTC cell lines. The five permanent CTC cell lines, namely BHGc7, 10, 16, 26 and UHGc5, express the typical SCLC markers, such as CD56/NCAM, enolase-2, chromogranin, synaptophysin and EpCAM (except UHGc5), as well as unique mutations of p53 [35, 43]. All CTC lines grow as loosely attached single cells and spontaneously form large TOS in suspension in regular tissue culture [43]. Spheroids have been demonstrated to enrich the CSC fraction and the CTC lines express components of the noncanonical WNT pathway and interact with macrophages, a typical feature of CSCs [45–47].

With the single exception of the SCLC NCI-H69 cell line, salinomycin has more pronounced cytotoxic effects against the SCLC CTC cell lines including BHGc16, BHGc16 TOS, BHGc26 and UHGc5 TOS. TOS show higher resistance to chemotherapeutics such as cisplatin, topotecan and epirubicin compared to the corresponding single cell suspensions [35]. However, in response to salinomycin, BHGc10 TOS and BHGc26 TOS were more resistant, whereas BHGc7 TOS and UHGc5 TOS were more sensitive than the related single cell suspensions. BHGc16 TOS showed no alteration in chemosensitivity to this drug compared to single cells. In general, SCLC CTC spheroids are not sensitized to salinomycin and the difference in chemosensitivity between single cells and aggregates is far lower than the 100fold disparity observed in breast cancer mammospheres and tumor cells exhibiting a CD44^+^/CD24^−^ CSC phenotype [29]. Cell death mechanisms induced by the ionophore salinomycin seem to be dependent on the impairment of mitochondrial function, generation of reactive oxygen species (ROS) and induction of apoptosis [29, 48–50]. The in vitro IC_50_ values of salinomycin vary between 0.3 and 10 μM, depending on the cell type used and treatment period [51, 52]. However, salinomycin is not suitable for clinical application and the pleiotropic effects may be linked to its direct effects on DNA through intercalation [53].

Niclosamide showed high cytotoxic activity against most SCLC lines with exception of GLC14 and GLC16. Among the SCLC CTC lines, BHGc7 TOS and UHGc5 TOS were more resistant to niclosamide than the corresponding single cell fractions in contrast to BHGc10 TOS, BHGc16 TOS and BHGc26 TOS which exhibited the reversed effect. Again, this CSC-selective drug exhibits heterogenous activity against the SCLC CTC spheroids. Niclosamide has antitumor activity via inhibition of NF-κB, an increase in ROS and blocking of the Wnt/β-catenin, STAT3 and NOTCH signaling pathways but also targets mitochondria in cancer cells [54, 55]. However, the multitude of activities of niclosamide may be explained by its function as nonselective DNA intercalator [56]. Niclosamide induces mitochondrial uncoupling, which renders mutant p53 cells, such as SCLC cells, susceptible to mitochondrial-dependent apoptosis [57, 58]. In conclusion, salinomycin and niclosamide lack consistent cytotoxic activity against the multicellular CTC TOS in contrast to CSC-enriched spheres of other tumor types.

SCLC is sensitive to the first-line therapy in most patients but invariably recurs as chemo- and radioresistant tumor within approximately one year [36]. This natural history of SCLC would fit a CSC model with elimination of the bulk tumor with initial cycles of chemotherapy and regrowth of the small population of resistant CSCs. Furthermore, the CSCs are hypothesized to be part of the large number of CTCs in SCLC and to constitute the true metastasis-inducing cells, as so-called circulating CSCs [7]. However, the present findings with the CSC-selective drugs salinomycin and niclosamide are not compatible with a consistent CSC phenotype of the SCLC cells and spheroids. Possible CSC markers described in SCLC cell line studies comprise CD44, CD90, CD133, CD87, Oct-4, SOX2, ALDH1, uPAR/CD87, CD166/ALCAM, BMI-1, PODXL-1, and PTCH [59]. CD44 plays an important role in tumor initiation and metastasis and seems to be involved in sphere formation in primary SCLC lines [13, 60]. Spheroid-forming cells were mainly found within the CD44^+^ CD90^+^ subpopulation expressing increased levels of Nanog and Oct-4. CD133 (Prominin 1) has been used to identify CSCs in lung cancer and was linked to high levels of ABCG2 and chemoresistance [61]. Several SCLC cell lines were found to contain a consistent side population (SP) fraction that comprises <1% of the bulk population [62, 63]. The SCLC cell line NCI-H446 exhibits a high degree of stemness, tumorigenicity and plasticity correlated to expression of CD133, Sall4, Oct-4, nestin, neural cell adhesion molecule (NCAM), S100β, vimentin, CD44 and CD105 [64]. CD133 positivity was described in H345 and NCI-H69 SCLC cell lines and the putative CSC markers urokinase plasminogen activator/receptor (uPA/uPAR CD87) are involved in cell migration, invasion and inhibition of apoptosis in SCLC CSCs [65, 66]. However, in a panel of six SCLC cell lines CD133^−^/CD87^−^ cells showed the highest tumorigenic potential [67]. Significant correlation between SOX2 mRNA expression levels and the number of distant metastatic sites was established in SCLC patients [68]. In conclusion, the correlation of the CSC phenotype in SCLC cells with markers previously established in other tumors is questionable and seems not to be of general validity.

In respect to CTCs, CD44 has been identified as CSC marker of gastric cancer and most of the CTC-positive patients showed CD44-positive CTCs [69]. In breast cancer patients, bone marrow metastases are enriched in CD44^high^/CD24^low^ cells, which have also been found in pleural metastases [70]. Recent studies have demonstrated that stem cell markers are frequently overexpressed in CTCs of breast cancer [40]. Early dissemination in SCLC is most likely related to the extremely high numbers of circulating tumor cells (CTCs) reaching counts of over several hundred CTCs per 7.5 ml blood compared to a 3–5 CTCs/7.5 ml as prognostic threshold for other solid tumors [71]. Our results demonstrate that SCLC tumor and CTC cells express CD133, but with exception of the BHGc10 CTC line obtained from a patient with primary resistance to cisplatin and GLC16, this antigen was mainly found in DMS53, GLC14 and NCI-H69 cell lines derived from patients prior to therapy [41, 42]. CD24 was co-expressed with CD133 in NCI-H69, DMS53, and GLC14 tumor cells and detectable in all CTC SCLC cell lines. In ovarian cancer, CD24^+^ CSCs were reported to be less proliferative and more resistant to chemotherapy compared to CD24^−^ cells [72]. With exception of NCI-H69, CD44 was low or absent from all CD133^+^ SCLC cells and, in particular, from all SCLC CTC lines. No expression of the chemoresistance mediator ABCG2 could be detected in the SCLC lines studied here (data not shown). In conclusion, CD133 is not expressed in response to prior chemotherapy and a CD24^+^/CD44^−^/ABCG2^−^ phenotype does not fit to the panel of markers generally expressed by typical CSCs of solid tumors, with the possible exception of BHGc10. Furthermore, SOX2 seems to represent a general marker of the neuroendocrine SCLC tumors which is not restricted to CSCs. Expression of Oct-3/4 and Nanog is limited to BHGc16 und BHGc26 which lack significant expression of CD133 and GLC14/16.

The numerous studies reporting CSC traits in SCLC tumor cells point to a possible role of this phenotype in resident SCLC primaries and secondaries but our results preclude a role of CSC characteristics among SCLC CTCs [73, 74]. It cannot be excluded that CSC plasticity allows for a switch to a non-CSC cell type in the circulation [6]. SCLC dissemination seems to be related to metastasis-initiating CTCs which exhibit a CD44^−^/CD24^+^/ABCG2^−^ salinomycin-sensitive phenotype which lack CD133 expression. With exception of SOX2, pluripotent stem cell transcription factors Oct-3/4 and Nanog are expressed to a minor extent in the CTC cell lines studied. The SCLC CTC cell lines are chemosensitive to topotecan and epirubicin as single suspensions in good agreement with the lack of ABCG2 expression [35]. However, TOS are chemoresistent and likewise radioresistant due to large size and hypoxic conditions in the core region [75, 76]. CSCs were reported to reside in hypoxic niches but SCLC CTCs recapitulate this strategy by the existence of quiescent and hypoxic cells in the TOS.
